# NAVIGATING DEMENTIA DURING COVID-19: THE EXPERIENCES OF GAY AND LESBIAN OLDER ADULTS

**DOI:** 10.1093/geroni/igac059.3034

**Published:** 2022-12-20

**Authors:** Laura Girling, Mike Splaine

**Affiliations:** Towson University, Towson, Maryland, United States; Splaine Consulting, Columbia, Maryland, United States

## Abstract

By 2030, it is estimated that 30 million individuals worldwide will have Alzheimer’s disease or related-dementias (hereafter dementia). Described as a “modern epidemic of later life,” dementia research has begun to reflect the diversity of our aging society with greater attention to minority populations. Nonetheless, some marginalized dementia-affected populations remain understudied. Estimates suggest more than 1 million lesbian, gay, bisexual, and transgender older adults will have dementia by 2030. Despite sizeable predictions, dementia-affected gay and lesbian populations remain critically understudied particularly in relation to COVID-19. To date, there is scarce literature focusing on how community-dwelling gay and lesbian adults with dementia navigate the management of their condition during the COVID-19 pandemic. In order to identify how community-dwelling gay and lesbian adults manage their dementia during the COVID-19 pandemic, data were combined from two interview-based studies. Content analysis was conducted on the interview narrative of the subset of individuals with dementia identifying as gay or lesbian and their study partner(n=18). Thematic findings include: 1.triple marginalization (age, sexual orientation, cognitive status), 2.social isolation, 3.programming effects (e.g., adult day programming, ADRD support groups), 4.informal and formal care interruptions, and 5.symptom acceleration. While all members of the public have experienced difficulties since the onset of COVID-19, health threats vary depending on social circumstances and vulnerabilities. Analyses indicate community-dwelling gay and lesbians with dementia are uniquely impacted by COVID-19 and face unique challenges. Attunement to such issues are of special interest to community-level policy makers, health providers, and older LGBTQ adults themselves, requiring further attention.

